# Supplementary material for: Prevalence of Bladder and Bowel Dysfunction in Duchenne Muscular Dystrophy Using the Childhood Bladder and Bowel Dysfunction Questionnaire
Source: Life (Basel). 2021 Jul 30;11(8):772. doi: 10.3390/life11080772 (PMC8399211; doi:10.3390/life11080772)
Supplement: Supplementary file 1 [file life-11-00772-s001.zip › life-1267481-S1_CBBDQ.pdf]

Childhood Bladder and Bowel Dysfunction Questionnaire (CBBDQ)

De vragenlijst voor plas- en ontlastingsproblemen bij kinderen van 5-12 jaar

Kies bij elke vraag het antwoord dat het beste van toepassing is op uw kind in de afgelopen maand.

Als u het antwoord niet weet, vraag het dan aan uw kind (of vul de vragenlijst samen met uw kind in).

NB. niet alle vragen hebben dezelfde antwoordmogelijkheden.

Mijn kind...

1	plast overdag vaker dan 8 keer.	☐ nooit	☐ 1 maal per maand of minder	☐ meerdere malen per maand	☐ 1 of meerdere malen per week	☐ (bijna) dagelijks
2	heeft overdag een natte (onder)broek (*een klein plekje wordt als nat beschouwd*).	☐ nooit	☐ 1 maal per maand of minder	☐ meerdere malen per maand	☐ 1 of meerdere malen per week	☐ (bijna) dagelijks
3	verliest direct na het plassen enkele druppels urine.	☐ nooit	☐ 1 maal per maand of minder	☐ meerdere malen per maand	☐ 1 of meerdere malen per week	☐ (bijna) dagelijks
4	verliest binnen één uur na het plassen urine.	☐ nooit	☐ 1 maal per maand of minder	☐ meerdere malen per maand	☐ 1 of meerdere malen per week	☐ (bijna) dagelijks
5	lijkt de aandrang tot plassen te negeren.	☐ nooit	☐ 1 maal per maand of minder	☐ meerdere malen per maand	☐ 1 of meerdere malen per week	☐ (bijna) dagelijks
6	gebruikt trucjes om droog te blijven, zoals wiebelen of benen stijf gekruist houden.	☐ nooit	☐ 1 maal per maand of minder	☐ meerdere malen per maand	☐ 1 of meerdere malen per week	☐ (bijna) dagelijks
7	wordt overvallen door een onbedwingbare aandrang om te plassen.	☐ nooit	☐ 1 maal per maand of minder	☐ meerdere malen per maand	☐ 1 of meerdere malen per week	☐ (bijna) dagelijks
8	stelt de eerste plas van de ochtend uit.	☐ nooit	☐ 1 maal per maand of minder	☐ meerdere malen per maand	☐ 1 of meerdere malen per week	☐ (bijna) dagelijks
9	plast tijdens de slaap in bed of luier.	☐ nooit	☐ minder dan 1 maal per week	☐ 1 tot 2 maal per week	☐ 3 tot 5 maal per week	☐ (bijna) dagelijks
10	wordt 's nachts wakker om te plassen.	☐ nooit	☐ minder dan 1 maal per week	☐ 1 tot 2 maal per week	☐ 3 tot 5 maal per week	☐ (bijna) dagelijks
11	heeft twee keer of minder per week ontlasting.	☐ nooit	☐ 1 maal per maand of minder	☐ hooguit 1-2 keer per maand	☐ meerdere malen per maand	☐ heel vaak
12	heeft ontlasting of vegen in de onderbroek.	☐ nooit	☐ 1 maal per maand of minder	☐ meerdere malen per maand	☐ 1 of meerdere malen per week	☐ (bijna) dagelijks
13	heeft harde poep of pijn bij de ontlasting.	☐ nooit	☐ 1 maal per maand of minder	☐ meerdere malen per maand	☐ 1 of meerdere malen per week	☐ (bijna) dagelijks
14	heeft een grote hoeveelheid ontlasting (*waarvan het toilet verstopt kan raken).*	☐ nooit	☐ 1 maal per maand of minder	☐ meerdere malen per maand	☐ 1 of meerdere malen per week	☐ (bijna) dagelijks
15	stelt het ontlasten uit.	☐ nooit	☐ 1 maal per maand of minder	☐ meerdere malen per maand	☐ 1 of meerdere malen per week	☐ (bijna) dagelijks
16	wordt overvallen door een onbedwingbare aandrang om te ontlasten.	☐ nooit	☐ 1 maal per maand of minder	☐ meerdere malen per maand	☐ 1 of meerdere malen per week	☐ (bijna) dagelijks
17	heeft buikpijn.	☐ nooit	☐ 1 maal per maand of minder	☐ meerdere malen per maand	☐ 1 of meerdere malen per week	☐ (bijna) dagelijks
18	heeft een opgeblazen buik.	☐ nooit	☐ 1 maal per maand of minder	☐ meerdere malen per maand	☐ 1 of meerdere malen per week	☐ (bijna) dagelijks
