# Supplementary material for: Prevalence of Bladder and Bowel Dysfunction in Duchenne Muscular Dystrophy Using the Childhood Bladder and Bowel Dysfunction Questionnaire
Source: Life (Basel). 2021 Jul 30;11(8):772. doi: 10.3390/life11080772 (PMC8399211; doi:10.3390/life11080772)
Supplement: Supplementary file 1 [file life-11-00772-s001.zip › life-1267481-S2_supplementary BBD questionnaire.pdf]

Maastricht UMC+

Centrum voor Neurologische Leer- en ontwikkelingsstoornissen
Kempenhaeghe

Codenummer: ………….

Aanvullende vragenlijst voor ouders van kinderen vanaf 6 jaar over mictie (plassen) en ontlasting (poepen)

Met deze vragenlijst proberen wij te weten te komen of uw kind problemen heeft met plassen. Onder plasproblemen/klachten verstaan wij: problemen bij de plas ophouden, erg vaak moeten plassen, niet op tijd voelen wanneer je moet plassen, verlies van plas zonder dat dit je bedoeling is, problemen bij het uitplassen. Ook willen we graag weten of uw kind problemen heeft met de ontlasting. Onder problemen met ontlastingen verstaan wij: ontlasting niet kunnen ophouden en obstipatieklachten.

Denkt u bij het beantwoorden van de vragen aan de klachten van de afgelopen 4 weken. Als er meerdere antwoorden op de situatie van uw kind van toepassing zijn, kies dan het antwoord wat u het beste vindt.

Wilt u per vraag één hokje aankruisen. Het zijn 33 meerkeuzevragen.

Algemeen

- Wat is de leeftijd van uw kind? ……. Jaar
- Heeft uw zoon naast de spierziekte ook andere medische problemen?
  - Nee
  - Ja, welke? …………………………………………
- Gebruikt uw kind medicijnen?
  - Nee
  - Ja, welke? …………………………………………………..
- Heeft uw kind wel eens een operatie gehad?
  - Nee
  - Ja, welke operatie(s)? ……………………………………..
- Gebruikt uw kind een rolstoel om zich voort te bewegen?
  - Nee
  - Ja
  - Anders, op welke manier? ……………………………………………
- Op wat voor school zit uw kind?
  - Reguliere school
  - Mythylschool
  - Anders, namelijk………………………………………………………
- Welk voortgezet onderwijs volgt uw kind of heeft hij gevolgd?
  - VMBO
  - HAVO
  - VWO
  - Anders, namelijk ……………………………………………………
- Is er in het verleden eens een psychologische en/of psychiatrische diagnose gesteld bij uw kind (bijvoorbeeld ADHD, autisme, dyslexie, depressie, angst, opstandig gedrag, obsessief gedrag)?
  - Nee

- Ja, welke diagnose? ……………………………………………..

Blaasproblemen en plasklachten

Zindelijkheid

1. Wanneer werd uw kind zindelijk voor urine overdag?
   - Leeftijd …………………………….………….jaar
- Nooit zindelijk geweest
- Ik weet het niet

2. Wanneer werd uw kind zindelijk voor urine s' nachts?
   - Leeftijd ……………………………………….jaar
- Nooit zindelijk geweest
- Ik weet niet

Algemene vragen over plassen

3. Als uw kind naar de wc gaat, moet hij dan wel eens wachten voordat de plas komt?
- Nooit
- Soms
- Vaak
- Altijd
- Ik weet het niet

4. Moet uw kind wel eens drukken of persen voor de plas komt?
- Nooit
- Soms
- Vaak
- Altijd
- Ik weet het niet

5. Als uw kind aan het plassen is, stopt dan wel eens onbedoeld het plassen?
- Nooit
- Soms
- Vaak
- Altijd
- Ik weet het niet

6. Heeft uw kind, tijdens of na het plassen, weleens last van een branderig (pijnlijk/vervelend) gevoel?
- Nooit
- Soms
- Vaak
- Altijd
- Ik weet het niet

7. Moet uw kind nadat hij net geplast heeft, wel eens direct erna weer plassen?
- Nooit
- Soms
- Vaak

- Altijd
- Ik weet het niet

8. Heeft uw kind een verminderde straal bij het plassen?
- Nooit
- Soms
- Vaak
- Altijd
- Ik weet het niet

Als u bij vraag 1 en 2 'nooit zindelijk geweest' heeft ingevuld en bij één of meer vragen uit vraag 3 t/m 8 'vaak' of 'altijd' heeft ingevuld, vult u dan onderstaande vragen 9 t/m 16 in over deze plasklachten.

Zo niet, gaat u dan door naar vraag 17.

Ontstaan van klachten

9. Wanneer had uw kind voor het eerst blaasproblemen of plasklachten?
   - Leeftijd ………………………………….……jaar
   - Welke klacht? ……………………………………………………………………………….

10. Heeft u een verklaring voor het ontstaan van de klachten? **(Hier zijn meerdere antwoorden mogelijk)**
- Pijn
- Angst
- Operatie. Welke?..
- Rolstoel afhankelijkheid
- Beademing

- Life event. Welke?...
- Ik weet het niet
- Anders, namelijk……………………………………………………………………………….

11. Is er een periode geweest dat de klachten verdwenen waren?
- Ja
- Nee
- Ik weet het niet

Als u bij vraag 11 'Ja' heeft ingevuld, vult u dan onderstaande vragen A. en B. in over deze plasklachten.

Zo niet, gaat u dan door naar vraag 12.

A. Zo ja, hoe lang duurde dat?
- ……………………………………………….…maanden
- Ik weet het niet

B. Zo ja, was dit na starten van een bepaalde behandeling?

- o Ja. Welke behandeling? …………………………………………………
- o Nee

12. Door wie werd uw kind tot nu toe behandeld voor deze klachten? **(Hier zijn meerdere antwoorden mogelijk)**

- o Huisarts
- o Kinderarts
- o Uroloog
- o Psycholoog/GGZ
- o Fysiotherapeut
- o Anders, namelijk………………………………………………………………

13. Gebruikt uw kind medicatie voor de blaasproblemen of plasklachten?

    - o Nee
    - o Ja.
    - \- Welke en in welke dosering en frequentie?..
    ……
- o Ik weet niet welke en hoeveel en hoe vaak.

Als u bij vraag 13 'Ja' heeft ingevuld, vult u dan onderstaande vragen A. en B. in over deze medicijnen.

Zo niet, gaat u dan door naar vraag 14.

A. Zo ja, hoe lang krijgt uw kind al medicatie voor de blaasproblemen of plasklachten?

\- ………………………………………………….maanden

- o Ik weet het niet

B. Was er sprake van verbetering na starten medicatie?

- o Nee
- o Ja
- o Ik weet het niet

14. Wat deed u zelf aan behandeling van deze klachten bij uw kind? **(Hier zijn meerdere antwoorden mogelijk)**

- o Beloning ('iets lekkers', cadeautje etc.)
- o Toilettraining
- o Bekkenbodemfysiotherapie
- o Anders, namelijk……………………………………………………………………………………………
- o Niet

15. Is er wel eens medisch onderzoek gedaan naar deze klachten?

- o Nee
- o Ja. Welk medisch onderzoek?...

16. _Is uw kind wel eens in het ziekenhuis opgenomen geweest vanwege dit probleem?

- o Nee

- o Ja.
  - Hoe vaak?...
  - Hoe oud was uw kind tijdens deze opnames?... jaar **(Hier mag u meerdere leeftijden aangeven)**

Problemen met ontlasting

Zindelijkheid

17. Wanneer werd uw kind zindelijk voor de ontlasting?
    - Leeftijd ………………………………………………………………….jaar
- o Nooit zindelijk geweest
- o Ik weet het niet.

Algemene vragen over de ontlasting

18. Heeft uw kind wel eens bloed bij de ontlasting?
- o Nee
- o Ja. Hoe vaak in de afgelopen maand? ……………………………………………………………………
- o Ik weet het niet

19. Heeft uw kind wel eens wondjes rondom de anus?
- o Nee
- o Ja
- o Ik weet het niet

20. Voelt uw kind de ontlasting aankomen?
- o Nee
- o Soms
- o Ja
- o Ik weet het niet

Als u bij vraag 17 'nooit zindelijk geweest' heeft ingevuld, bij vraag 18 en 19 'ja' en bij vraag 20 'nee', vult u dan onderstaande vragen 21 t/m 28 in over deze problemen met ontlasting.

Zo niet, gaat u dan door naar vraag 29.

Ontstaan van klachten

21. Wanneer had uw kind voor het eerst obstipatieklachten of vieze broeken?
    - Leeftijd ……………………………………………………………………………………..………………jaar
    - Welke klacht? ………………………………………………………………………………………………..
22. Heeft u een verklaring voor het ontstaan van de klachten? **(Hier zijn meerdere antwoorden mogelijk)**
- o Pijn
- o Angst
- o Operatie. Welke?...
- o Rolstoel afhankelijkheid
- o Beademing
- o Life event. Welke?..

- Ik weet het niet
- Anders, namelijk……………………………………………………………………………………………..

23. Is er een periode geweest dat de klachten verdwenen waren?
- Ja
- Nee
- Ik weet het niet

Als u bij vraag 23 'Ja' heeft ingevuld, vult u dan onderstaande vragen A. en B. in over deze poepklachten.

Zo niet, gaat u dan door naar vraag 24.

A. Zo ja, hoe lang duurde dat?
- ……………………………………………………………………………….maanden
- Ik weet het niet

B. Zo ja, was dit na starten van een bepaalde behandeling?
- Ja. Welke behandeling? ……………………………………………………………………………………………
- Nee

24. Door wie werd uw kind tot nu toe behandeld voor deze klachten? **(Hier zijn meerdere antwoorden mogelijk)**
- Huisarts
- Kinderarts
- Psycholoog/GGZ
- Fysiotherapeut
- Anders, namelijk……………………………………………………………………………………………………

25. Gebruikt uw kind medicatie voor de ontlasting?
- Nee
- Ja.
  - Welke en in welke dosering en frequentie?...
    ……
- Ik weet niet welke en in welke dosering en frequentie

Als u bij vraag 25 'Ja' heeft ingevuld, vult u dan onderstaande vragen A. en B. in over deze medicijnen.

Zo niet, gaat u dan door naar vraag 26.

A. Zo ja, hoe lang krijgt uw kind al medicatie voor de ontlasting?
- …………………………………………………………………………………………………..maanden
- Ik weet het niet

B. Was er sprake van verbetering na starten medicatie?
- Nee
- Ja

- Ik weet het niet

26. Wat deed u zelf aan behandeling van deze klachten bij uw kind? **(Hier zijn meerdere antwoorden mogelijk)**

- Vezelrijk dieet
- Beloning (‘iets lekkers’, cadeautje etc.)
- Toilettraining
- Bekkenbodemfysiotherapie
- Anders, namelijk……………………………………………………………………………………………….
- Niet

27. Is er wel eens medisch onderzoek gedaan naar deze klachten?
    - Nee
    - Ja. Welk medisch onderzoek?...

28. Is uw kind wel eens in het ziekenhuis opgenomen geweest vanwege dit probleem?

- Nee
- Ja.
    - Hoe vaak?...
    - Hoe oud was uw kind tijdens deze opnames?... jaar **(Hier mag u meerdere leeftijden aangeven)**

Overig

Manier van naar de wc gaan voor zowel mictie als ontlasting

29. Gebruikt uw kind bij het naar de wc gaan een hulpmiddel?

- Nee
- Ja. Welke?………………………………………………………………………………………………….
-

30. Heeft uw kind een aangepast toilet nodig?

- Nee
- Ja. Welke aanpassingen zijn er nodig?...
  ……

31. Kan uw kind zelfstandig naar het toilet?

- Nee. Waar is hulp bij nodig?...
  ………..
- Ja

Combinatie patroon

32. Ziet u een relatie tussen de plasklachten en obstipatieklachten?

- Nee
- Ja. Welke relatie?...
  ………..
  ……….
- Ik weet het niet

Invloed op dagelijks leven

33. Indien uw kind last heeft van een van de blaas- of darmproblemen die in de vragenlijst genoemd worden, in hoeverre wordt uw kind hierdoor beperkt in de dagelijkse bezigheden op schaal van 0-10 (0= geen beperking; 10 = ergst mogelijke beperking)?

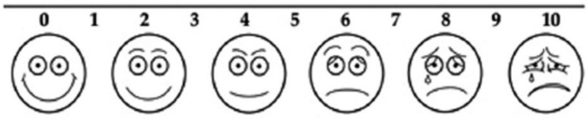

(Hier mag u het getal omcirkelen dat het beste past volgens u)

- Van welke klachten heeft uw kind last bij je dagelijkse bezigheden? ....
  …………………………………………………………………………………………………….
  ……………………………………………………………………………………………………

o Nee, mijn kind heeft geen heb geen plas- of poepklachten.

DANK VOOR UW MEDEWERKING
